# Shaping sustainable futures: The role of micro-entrepreneurs’ personality traits in social innovations

**DOI:** 10.1371/journal.pone.0306800

**Published:** 2024-08-14

**Authors:** Maciej Zastempowski

**Affiliations:** Department of Enterprise Management, Nicolaus Copernicus University, Torun, Poland; Kırklareli University: Kirklareli Universitesi, TÜRKIYE

## Abstract

In the rapidly evolving business landscape, micro-entrepreneurs stand out as significant contributors to social innovation. However, the link between their personality traits and the social innovations they introduce needs to be studied more. This research, guided by the Big Five model and the Oslo Manual’s innovation framework, aims to address this gap. The central question driving this study is whether the personality traits of micro-entrepreneurs, precisely openness to experience, conscientiousness, extraversion, agreeableness, and neuroticism, influence the social innovations they bring forth. Through a comprehensive exploration of literature and empirical analysis—quantitative research on a representative sample of 1848 Polish micro-entrepreneurs—this research examines the interconnectedness between personality characteristics and social innovation outcomes. The key findings suggest that three personality variables characterising micro-entrepreneurs–openness for experience, conscientiousness, and extroversion–emerge as shared, statistically significant factors. These variables positively impact all types of social innovations implemented by micro-entrepreneurs (product and process). In none of the analysed cases, agreeableness and neuroticism were statistically significant. Finally, it’s worth emphasising that the chances of micro-entrepreneurs introducing social innovations increase more strongly with an increase in their openness to experience than in the case of extroversion and conscientiousness.

## 1. Introduction

In today’s dynamic and ever-changing business environment [1], micro-entrepreneurs’ role in shaping social innovations is becoming increasingly prominent. Micro-entrepreneurs, constituting one of the fundamental groups of entrepreneurs, appear to play a crucial role in creating innovative solutions [2, 3], both at the product and business process levels [4]. Through the lens of Schumpeterian theory, they are the primary agents of creative destruction, contributing to economic development [5]. However, intriguingly, micro-enterprises still remain at the periphery of research in innovation [3]. Only a few studies attempt to shed light on this issue [6–9].

It is worth emphasising that, with the development of society, growing social challenges require increasingly innovative solutions [10, 11]. This has been globally recognised, indicating that social innovations, supporting the transformation of societies towards sustainable development [12, 13], play a significant role in achieving the Sustainable Development Goals (SDGs) set by the United Nations [14]. Micro-entrepreneurs are increasingly becoming key players in shaping these processes [15, 16]. Their flexibility, risk-taking ability, and proximity to local communities [17] make them ideally positioned to recognise and solve social problems at the grassroots level.

Looking from the perspective of the dominant role of the owner in micro-enterprises [17], it is worth considering whether the personality traits they exhibit translate into their innovativeness. The role of personality in the context of various aspects of innovation has already been the subject of numerous scientific works [18–24]. Within the field of psychology, various theories have emerged, including psychodynamic [25], cognitive-behavioural [26], humanistic [27], and trait-based [28] approaches. Particularly, the latter, aiming to identify key personality traits to predict future behaviour, seems crucial in the context of social innovation. Based on the widely accepted Big Five model [29, 30], which focuses on five major personality traits: openness to experience, conscientiousness, extraversion, agreeableness, and neuroticism (OCEAN), the question arises: *Can the social innovations introduced by micro-entrepreneurs be influenced by their personality traits*?

Attempting to answer this research question, we examined a representative sample of 1848 Polish micro-entrepreneurs in terms of their personality traits using the Big Five model [31] and the social innovations they implemented, viewed from the perspective of the OLSO methodology, indicating two types of innovations—product and business process [4].

The article is divided into several sections. Section two, dedicated to theoretical background, will take a closer look at the concept of personality, with particular emphasis on the Big Five model, social innovations, and the connections between these constructs. Section three presents the research methodology, describing the research sample, the measurement of variables, and the methods used to analyse the data. Section four presents the research results, and section five focuses on the discussion, interpretation, and conclusions drawn from the analysis.

## 2. Theoretical background

### 2.1 Concept of personality

The theoretical background in personality concepts encompasses various approaches that have shaped our understanding of human psychology over the years. Among these theories are notable perspectives such as the psychodynamic [25, 32, 33], the cognitive-behavioural [26, 34, 35], the humanistic [27, 36] and the trait-based [28, 37, 38]. Each of these theories emphasises different aspects of personality formation and functioning.

A psychodynamic perspective, originating in psychoanalysis developed by Freud, focuses on the role of unconscious mental processes, inner conflicts, and developmental stages in shaping personality [25]. The cognitive-behavioural approach concentrates on observable aspects of behaviour, learning processes, and interactions with the environment as critical elements influencing personality [39]. In contrast to psychodynamic or humanistic approaches, which emphasise mental processes and internal conflicts, the cognitive-behavioural approach concentrates on measuring, observing, and modifying specific behaviours. On the other hand, humanistic psychology, as represented by Rogers, underscores individual autonomy, the pursuit of self-actualisation, and personal development through cognitive and emotional experiences [27]. The trait-based approach in personality theory focuses on identifying and describing fundamental traits that characterise individuals [28, 37]. This approach enables the study and description of individual differences in a more structured manner, contributing to a better understanding of how personality traits impact an individual’s behaviour and functioning in different contexts.

Despite the richness of these theories and related models for examining human personality, such as Cattell’s Sixteen Personality Factor Questionnaire [40], Eysenck’s Three-Factor Model [41], Myers-Briggs Type Indicator [42] or HEXACO model [43], one of the most influential and widely applied models is the Five-Factor Model, also known as the Big Five. Developed by several research groups [29, 30, 44, 45], this model posits the existence of five major personality traits: openness to experience, conscientiousness, extraversion, agreeableness, and neuroticism. It is worth emphasising that this model has gained recognition due to several features, including strong empirical bases, comprehensiveness and simplicity, predictive power, universality, applicability across cultures, and practical utility in personality research [19, 46–49]. Below, there is a concise overview of the Big Five traits.

*Openness to experience* is a trait that reflects an individual’s willingness to explore, originality in thinking, and openness to various life experiences [48, 50]. Individuals with high levels of openness are typically creative, curious about the world, and ready for new challenges [38, 51, 52]. They have an affinity for art, abstract ideas, and diverse cultures [53]. They possess the ability for unconventional thinking, are flexible in problem-solving, and are open to diverse perspectives [50]. Conversely, individuals with lower levels of openness may prefer tradition, routine, and familiar patterns, influencing a more conservative approach to life [29].

*Conscientiousness* is a trait that characterises the level of organisation, meticulousness, and self-discipline in one’s approach to life [48]. Conscientious individuals are like architects of their destiny, precisely planning their actions and striving to achieve set goals. They engage wholeheartedly in their duties, pay attention to details, and aim for perfection [54]. Conscientiousness influences effectiveness in tasks both in the professional and personal spheres. In interpersonal relationships, conscientious individuals are considered reliable and trustworthy [55]. Increased conscientiousness levels are associated with individuals who are reliable, driven, and meticulous, as indicated by McCrae and Costa [56], and who prioritise deliberate actions over spontaneous ones, as suggested by Barrick and Mount [54] and Feist [52].

*Extraversion* is a trait that describes an individual’s inclination toward sociability, assertiveness, and social activity [47]. Individuals with high levels of extraversion are like socialites, drawing energy from interactions with other people [20]. They are sociable, confident, easily establish connections, and are socially active. In social relationships, they are open, friendly, and easily build lasting interpersonal bonds [57]. On the other hand, individuals with low levels of extraversion may prefer solitude, avoid intense social interactions, and focus more on their own thoughts and feelings [56].

*Agreeableness* is a trait that characterises an individual’s tendency toward kindness, cooperation, and avoiding conflicts [58]. Agreeable individuals are like life diplomats, ready to cooperate and always striving to maintain harmony in relationships [56]. They are empathetic individuals who are willing to help others and have a high ability to understand other people’s perspectives [48, 50]. In group settings, they serve as connectors, aiming to maintain a positive atmosphere and resolve conflicts. A high level of agreeableness suggests a tolerant, friendly, polite, well-disposed, trusting and helpful person [56, 59]. Individuals with low levels of agreeableness may be more decisive, assertive, and inclined to express their opinions [52, 56].

*Neuroticism* is a trait that indicates an individual’s inclination to experience negative emotions such as anxiety, unease, or depression [56, 60]. Neurotic individuals are like emotional thermometers, reacting intensely to stressful life situations [53]. They tend to experience emotions on a profound level, which can impact their overall well-being [20]. Neurotic individuals are often more sensitive to changes in their environment and may struggle with coping in situations requiring emotional regulation [58]. However, neuroticism can also contribute to deeper introspection and the development of coping strategies for emotional difficulties [19, 56].

It is essential to highlight that despite ongoing discussions regarding the appropriateness of the Big Five model [61–63], the literature offers multiple compelling pieces of evidence supporting its resilience [29, 64]. Prior investigations have not only demonstrated the consistency of the Big Five as personality traits across various cultures [65] but have also suggested their hereditary nature [45] and stability over time [66].

### 2.2. Social innovation

In recent decades, the evolution of the concept of social innovation has given rise to a multifaceted understanding, capturing the attention of scholars and practitioners alike [67–70]. A plethora of perspectives have emerged, revealing the complexity of social innovation [71–76]. At its core, social innovation is a dynamic and adaptive process that involves the generation and implementation of novel solutions to address pressing societal challenges while fostering positive societal change [14].

Social innovations play a significant role in addressing various social issues and improving people’s quality of life. They are also progressively acknowledged as catalysts for promoting sustainable development and fostering smart and inclusive growth [72, 77]. Their significance extends to various fields, including education [78, 79], healthcare [80, 81], environment [82], employment [70], social integration [83, 84], and many other areas [69, 85, 86].

One prominent characteristic of social innovations is their transformative nature. Unlike incremental changes, social innovations often bring about systemic shifts, reshaping existing paradigms and structures [72, 77]. This transformative aspect is particularly crucial in addressing complex issues such as poverty, inequality, and environmental degradation, where conventional approaches may fall short [14].

Furthermore, social innovations are frequently characterised by their participatory and collaborative nature. Engaging diverse stakeholders, including communities, NGOs, businesses, and governments, is central to the success of social innovation initiatives [87]. Co-creation and co-design are integral components, ensuring that solutions are contextually relevant and inclusive.

It is also worth sharing the opinion of Oliinyk et al., indicating that social responsibility (a concept that includes, among others, social innovation) should be perceived not only as an important socially oriented concept but also as a reliable basis for the development of innovative business, primarily thanks to the creation of a comfortable institutional environment for business development [88].

Drawing from theories such as the diffusion of innovations [89] and the social construction of technology [90], social innovations are seen as spreading through networks and social structures. The diffusion theory posits that innovations pass through awareness, adoption, and implementation stages within a social system, highlighting the importance of communication channels and influential actors in the process [89]. Meanwhile, the social construction of technology underscores the sociocultural context in which innovations are developed, emphasising the interplay between technology and societal values [90, 91].

Importantly, the concept of social innovation intersects with the broader landscape of innovation ecosystems [92]. Innovation ecosystems encompass a network of actors, resources, and institutions collectively contributing to the innovation process [93–95]. Within the social innovation context, these ecosystems involve collaboration across sectors, with social enterprises, academia, and governmental bodies playing pivotal roles [93].

In summary, social innovation is a dynamic and transformative process characterised by participatory approaches, diffusion through social networks, and integration within broader innovation ecosystems. Considering the above, it was decided to understand social innovations in two dimensions, in accordance with the concept of the OSLO Manual [4], namely as social product and process innovations that improve the quality of life of people, communities and nations [87].

### 2.3. Links between personality traits and social innovations

Past investigations across various fields have revealed that one can employ consistent individual characteristics to recognise individuals exhibiting creativity and innovation in their actions [19, 96, 97]. However, the results are not definitively conclusive [98], prompting Mendoza-Silva to advocate further exploration into how personality traits influence innovation capability, as well as in the field of social innovation, given the substantial gap in existing research on this matter [99].

Based on our understanding, which stems from a systematic literature review utilising the Web of Science and Scopus databases, more evidence is needed to validate the connection between the Big Five personality traits and social innovation. Next, we present available research results indicating various relationships between the Big Five personality traits and other various aspects of innovation.

Research conducted in the domain of openness to experience has indicated a positive relationship between openness to experience and the innovativeness of engineers [18], the personal innovativeness of students [19], individual innovation competencies [20], innovative work behaviour [100], innovation capability [21], and national levels of innovation [101].

The available literature also supports the favourable connection between conscientiousness and the innovativeness of engineers [18], the personal innovativeness of students [19], individual innovation competencies [20], the success of radical new product development teams [102], the prolonged survival of ventures [103], and innovation capability [21].

Additionally, a favourable relationship emerged between extroversion and the innovativeness of engineers [18], the personal innovativeness of students [19], individual innovation competencies [20], innovation capability [21], and innovations developed through the closed doing-using-interacting mode [104].

Earlier research has also indicated a positive relationship between agreeableness and the personal innovativeness of students [19], technological innovation [21], and innovation levels at a national scale [101]. It is also worth pointing out that one of the key aspects of agreeableness, in addition to compassion, kindness, willingness to cooperate and empathy, is trust. In this area, the results of earlier research indicate the existence of a relationship between inter-organisational trust, innovation and financial performance [105]. Conversely, findings presented by Abdullah et al. propose an adverse relationship, where elevated levels of agreeableness lead to reduced creativity [46].

In turn, in the area of neuroticism, earlier studies have proposed an adverse correlation between neuroticism and the innovativeness of engineers [18], the personal innovativeness of students [19], creativity [46], the success of both radical and incremental new product development teams [102], innovative performance [106], and innovations generated through the open doing-using-interacting mode [104].

Considering the aforementioned, it is valuable to articulate the following research question: *Can the social innovations introduced by micro-entrepreneurs be influenced by their personality traits*?

## 3. Methodology

Adopting Comte’s positivist approach, focusing on the scientific pursuit of knowledge of reality [107] and falling within the paradigm of Burrell and Morgan’s functionalism [108], various methods were used to try to obtain an answer to the formulated research question. The presented theoretical background uses a narrative synthesis as part of a systematic literature review [109] based on two bibliometric databases—Web of Science and Scopus. In the empirical part, data were obtained by indirect survey measurement in the form of computer-assisted telephone interviewing (CATI), using measurement scales validated in previous studies. To ensure objectivity, the research was conducted on a randomly selected representative research sample, and the obtained microdata were analysed and assessed using logistic regression. Details regarding data gathering, measurement of variables and the statistical methods used are presented in the following sections.

### 3.1. Data gathering

The study focused on Polish micro-entrepreneurs, which is in line with the European Commission’s recommendation and includes enterprises employing up to 9 people [110]. Data collection took place from August to October 2022. To ensure the representativeness of the collected micro-data, a random sample of micro-entrepreneurs was selected from the National Official Register of Economic Entities (NOREE) in collaboration with the Statistical Office of Poland. The sampling frame comprised active micro-enterprises, numbering 4497099 in Poland in 2022. Stratified sampling was applied, with layers distinguished based on the type of activity, administrative region, and legal form. The primary sample of 1850 units was allocated proportionally to the size of each layer in the sampling frame. Additionally, a separate reserve sample was drawn, matching the structure and size of the primary sample, which was 19 times larger. The total number of randomly drawn samples, including primary and reserve samples, amounted to 36994. The final dataset covered 1848 micro-enterprises, allowing for concluding with a maximum error of +/- 3% at a confidence level of 99%, considering a fraction size of 50%.

### 3.2. Variable measurement

The evaluation of social innovativeness among micro-entrepreneurs, serving as the dependent variable, followed the guidelines presented in the fourth edition of the OSLO Manual [4]. This involved distinguishing between product and business process innovations, alongside incorporating the European Commission’s perspective on comprehending social innovations [87].

In light of this, micro-entrepreneurs were queried—*Were any of the product or business process innovations introduced in 2019–2022 implemented for social purposes–improving the quality of life of people*, *communities*, *and nations*? Consequently, three binary variables were established:

- y_1_ –implementation of social product innovation,
- y_2_ –implementation of social business process innovations,
- y_3_ –implementation of both social product and business process innovations.

The investigation into the characteristics of micro-entrepreneurs utilised the Big Five Inventory, developed by John and Srivastava in 1999 [31], to assess personality traits, serving as the independent variables. This inventory comprises 44 statements evaluated on a five-point Likert scale. It is available in open access in the work of Jirasek and Sudzina [111]. Each personality trait was assigned a code:

- O–openness to experience,
- C–conscientiousness,
- E–extroversion,
- A–agreeableness,
- N–neuroticism.

The means of the respective items were employed to obtain final measurement values for the independent variables.

Additionally, based on previous research [112, 113], the following two control variables were included:

- Age—the period since the inception of the micro-entrepreneur’s venture, expressed numerically, with logarithmic adjustments incorporated into the calculations;
- Size—the scale of the micro-entrepreneur’s dimensions measured by the count of employees.

Descriptive statistics of the analysed variables are presented in Table 1.

**Table 1:** Variables’ descriptive statistics (N = 1848).

Variable	%—yes	Cronbach’s *α*	Mean	S.E.	M	D	S.D.	SD^2^	Min.	Max.
y_1_	4.978	-	0.050	0.005	0.000	0.000	0.218	0.047	0.000	1.000
y_2_	4.978	-	0.050	0.005	0.000	0.000	0.218	0.047	0.000	1.000
y_3_	3.571	-	0.036	0.004	0.000	0.000	0.186	0.034	0.000	1.000
O	-	0.806	3.538	0.014	3.500	3.500	0.616	0.379	1.400	5.000
C	-	0.905	3.750	0.018	4.000	4.556	0.788	0.621	1.222	4.889
E	-	0.856	3.555	0.019	3.625	4.250	0.804	0.647	1.250	4.750
A	-	0.938	3.293	0.021	3.333	2.667	0.892	0.795	1.333	5.000
N	-	0.875	2.682	0.021	2.625	3.000	0.888	0.789	1.000	5.000
Age	-	-	0.969	0.008	1.000	0.602	0.354	0.125	0.000	2.021
Size	-	-	2.692	0.068	2.000	0.000	2.937	8.628	0.000	9.000

### 3.3. Method

In our research, we examined a group of micro-entrepreneurs. However, our study involved human participants since we asked managers to answer survey questions. We informed participants that the survey was anonymous. Only adults participated in the study. All respondents gave their informed consent (oral) to participate in the study. We analysed data anonymously and did not ask about personal information. Therefore, our research, by the recommendations of the National Science Centre [93], which are the basis for drawing up guidelines for conducting research at our university [94], did not require the approval of ethics committees.

Since the dependent variables are binomial, a logistic regression model was used to analyse the relationship between the personality of a micro-entrepreneur and the introduction of social innovations. The logistic regression model can be written as follows:

$$logit(p_{i})=Z_{i}=x_{i}^{\prime }\beta =\beta_{0}+\beta_{1}X_{1i}+\beta_{2}X_{2i}+\cdots +\beta_{k}X_{ki}$$ (1)

whereas *logit* (*p*_*i*_) is denoted $\ln\frac{p_{i}}{1-p_{i}}$. The subject of estimation in this model are the parameters *β*_0_, *β*_1_, *β*_2_,…,*β*_*k*_ being elements of the vector *β*.

To interpret the results of the logit model estimation, odds ratios (OR) were used. If the likelihood is denoted as:

$$\frac{p_{i}}{1-p_{i}}=\exp\left(x_{i}^{\prime }\beta )=exp(\beta_{0}+\beta_{1}X_{1i}+\beta_{2}X_{2i}+\cdots +\beta_{k}X_{ki}\right)=\Omega (x_{i}),$$ (2)

then the odds ratios with the variable *X*_*mi*_ increased by a unit, and the odds without this increase equal:

$$\frac{\Omega (x_{i}^{m},X_{mi}+1)}{\Omega (x_{i}^{m},X_{mi})}=exp(\beta_{m}),$$ (3)

where $x_{i}^{m}$ is the vector *x*_*i*_ without the variable *X*_*mi*_. Formula (3) shows that the increase in the value of *X*_*mi*_ by one unit is related, *ceteris paribus*, with an *exp*(*β*_*m*_)-fold change in the odds ratio. In the case of *exp*(*β*_*m*_)>1 there is an increase, and in the case of *exp*(*β*_*m*_)<1 there is a decrease in the odds ratio.

In the estimation, we used the maximum likelihood method and STATA.16.1 software.

## 4. Results

To assess the potential presence of common method bias (CMB), Harman’s single-factor test [114] was employed, encompassing the Big Five variables scrutinised in the study. The outcome indicated that the exploratory single-factor model explained 29.72% of the variance, suggesting an absence of CMB concerns.

Sample adequacy for the Big Five variables was verified using the Kaiser–Meyer–Olkin (KMO) and Bartlett sphericity tests [115]. The KMO yielded a result of 0.814, and the Bartlett sphericity test (χ2 = 111840.050; df = 946) revealed a significance level below 0.001, which confirmed the reliability of the measurement scale. This was also confirmed by Cronbach’s alpha results, shown in Table 1.

The observations derived from the Kendall correlation coefficients in Table 2 offer several insights. Initially, a positive correlation exists between the two types of social innovations (y1 –y_2_) implemented by micro-entrepreneurs. Secondly, statistically significant coefficients are evident between the dependent and independent variables. However, the values for the Big Five personality traits consistently remain below 0.168, indicating a very weak interdependence. Thirdly, the coefficients among the Big Five personality variables consistently fall below 0.438, and all Variance Inflation Factors (VIF) are below 10 (with the highest observed VIF being 2.98), suggesting that multicollinearity is not a concern.

**Table 2:** Correlation matrix.

	y_1_	y_2_	y_3_	O	C	E	A	N	Age	Size
y_1_	1.000									
y_2_	0.703**	1.000								
y_3_	0.841**	0.841**	1.000							
O	0.167**	0.167**	0.146**	1.000						
C	0.148**	0.134**	0.117**	0.316**	1.000					
E	0.157**	0.168**	0.135**	0.316**	0.216**	1.000				
A	0.094**	0.109**	0.088**	0.149**	0.003	0.438**	1.000			
N	-0.073**	-0.098**	-0.072**	-0.187**	-0.034*	-0.409**	-0.627**	1.000		
Age	0.000	0.003	0.009	-0.004	0.030	-0.006	-0.027	0.014	1.000	
Size	-0.005	0.002	-0.013	-0.036*	-0.067**	0.098**	0.157**	-0.149**	-0.031	1.000

* p-Value ≤ 0.05, ** p-Value ≤ 0.01.

The results for the overall logistic estimations and odds ratios (OR) for social product innovation (y_1_), social business process innovations (y_2_), and both types of innovations together (y_3_) are shown in Table 3.

**Table 3:** Logistic regression.

	y_1_			y_2_				y_3_		
	Coef.	S.E.	OR	Coef.		S.E.	OR	Coef.	S.E.	OR
O	0.999**	0.240	2.718**	1.001**		0.251	2.722**	1.101**	0.287	3.009**
C	0.807**	0.198	2.243**	0.603**		0.190	1.829**	0.665**	0.215	1.946**
E	0.870**	0.245	2.388**	0.983**		0.239	2.674**	0.837**	0.280	2.310**
A	0.257	0.262	1.293	0.105		0.232	1.111	0.276	0.263	1.318
N	0.425	0.254	1.531	0.168		0.256	1.183	0.375	0.282	1.455
Age	0.196	0.298	1.211	0.179		0.312	1.196	0.405	0.352	1.499
Size	0.055	0.037	1.056	0.055		0.037	1.057	0.054	0.044	1.055
Cons_	-15.756**	2.081	1.444**	-14.189**		1.989	1.311**	-15.955**	2.206	1.188**
*Log pseudolikelihood*	-304.347			-304.108				-239.639		
*Wald chi*^*2*^ *(7)*	91.12			112.19				76.06		
*Prob > chi* ^*2*^	0.0000			0.0000				0.0000		
*Pseudo R*^*2*^ *McFadden*	0.1677			0.1684				0.1584		

* p-value ≤ 0.05, ** p-value ≤ 0.01. *Note*: Robust standard error in S.E. column. N = 1848

At a 0.01 confidence level, three personality variables characterising micro-entrepreneurs–namely, openness for experience (O), conscientiousness (C), and extroversion (E)–emerge as shared, statistically significant factors. These variables exhibit a positive impact on all types of social innovations implemented by micro-entrepreneurs (y_1_, y_2_, and y_3_). In none of the analysed cases, agreeableness (A) and neuroticism (N) turned out to be statistically significant. Based on the odds ratios (OR), it is clearly visible that the chances of microentrepreneurs introducing social innovations increase most strongly with an increase in their openness to experience, then in the case of extroversion and conscientiousness.

## 5. Discussion

The results obtained lead to several thoughts. First, openness to experience (O), a prominent personality trait within the framework of the Big Five model, pertains to an individual’s readiness to explore new ideas, experiences, and perspectives [48, 50]. In the context of micro-entrepreneurs, research indicates a significant positive influence of openness to experience to the ability to introduce social innovations, encompassing both product and business process domains (y_1_, y_2_, and y_3_). This result is consistent with previous research on the influence of personality on other aspects of innovation [18–21].

One key aspect that could elucidate this relationship is the ability of micro-entrepreneurs to adapt to a dynamic environment [17, 116]. Openness to experience is closely linked with cognitive flexibility and a willingness to embrace new ideas [38, 51]. Micro-entrepreneurs with a high level of openness are more inclined to accept new trends, technologies, and societal expectations. Consequently, they exhibit a greater readiness to experiment with novel products and implement changes in business processes.

Openness to experience enables micro-entrepreneurs to perceive their environment as a source of inspiration rather than a challenge or threat. This ability to positively perceive change and novelty becomes a pivotal factor conducive to generating social innovations. In the realm of products, micro-entrepreneurs with high openness are more disposed to create unique, surprising solutions that address genuine societal needs.

Furthermore, openness to experience is also associated with the ability to learn and adapt to new situations [117]. Micro-entrepreneurs with these personality traits are more open to gathering information, seeking knowledge, and adjusting to changing market conditions. Consequently, they can better comprehend the expectations of their customers, anticipate market trends, and tailor their products or services to current societal needs.

Another facet is the ability to build social relationships [56]. Micro-entrepreneurs with high openness to experience are often more willing to establish cooperation and partnerships. In the context of social innovations, where collaboration with local communities, non-governmental organisations, or public institutions is crucial [93, 95, 118], this personality trait can play a significant role. The ability to build relationships facilitates the collection of feedback, engages the local community in the innovation creation process, and secures support for socially innovative projects.

It is also worth noting that openness to experience is associated with actively seeking new cultural, artistic, and intellectual experiences [53]. Micro-entrepreneurs with this personality trait often draw inspiration from various sources, leading to the creation of innovative products inspired by different facets of life. The creativity stemming from openness to experience thus serves as a robust impetus for generating socially innovative solutions in micro-entrepreneurship.

Second, conscientiousness (C), also referred to as the degree to which an individual is organised, goal-oriented, inclined towards planning, and maintains discipline [48], has proven to have a significant positive influence on all types of social innovations introduced by micro-entrepreneurs (y_1_, y_2_, and y_3_). This result also refers to previous research in terms of the relationship between personality and innovativeness [18, 20, 21].

One of the key aspects explaining this relationship is the diligence and systematic approach of micro-entrepreneurs with high conscientiousness. Individuals with these traits exhibit a strong ability to plan and are meticulous in task execution [54]. In the context of social innovations, where the introduction of innovative solutions often requires precise planning and consistent implementation, conscientious micro-entrepreneurs find themselves in an advantageous position.

A high level of conscientiousness also translates into responsibility for one’s actions [56]. Micro-entrepreneurs who adopt a conscientious and responsible approach to business are more inclined to consider social and environmental aspects in their operations. Consequently, they are more open to implementing social innovations that often bring benefits to both society and the environment.

Moreover, conscientious micro-entrepreneurs are typically more inclined to conduct market research and analyse societal needs. Attention to detail and meticulousness in processing information [54] allow them to accurately identify existing gaps or social issues. These skills are crucial in the process of creating social innovations, which typically emerge as a response to real needs within the community [14].

Focusing on the realm of products, micro-entrepreneurs with high conscientiousness are more likely to produce goods or provide services that bring social benefits. These may include eco-friendly products, sustainable offerings, or those manufactured according to ethical labour standards [119]. Conscientiousness introduces an element of social values into the operations of micro-enterprises, thereby fostering the development of social innovations.

In the context of business processes, conscientiousness influences meticulous planning and resource management. Micro-entrepreneurs with this personality trait are more willing to implement effective management procedures, which can facilitate the introduction of social innovations in areas such as organisational work or supply chains. A conscientiously managed enterprise is better positioned to adapt to changing socio-economic conditions, a crucial context for social innovations.

High conscientiousness is also associated with building lasting relationships with stakeholders [55]. Micro-entrepreneurs with a conscientious approach to business often enjoy greater trust from the local community, customers, and business partners. This trust can be pivotal in the effective implementation of social innovations, which often require collaboration and acceptance from the local community.

Third, extraversion is the last personality trait that has proven to be significant in the context of social innovations implemented by micro-entrepreneurs. This result also refers to previous research on other aspects of innovation [18, 20, 104].

This relationship can be justified through various aspects of extraversion that interact with the dynamic and interactive nature of entrepreneurial activities, creating an environment conducive to generating and implementing social innovations.

Extraverts are characterised by ease in forming social relationships and a strong communication ability [20]. In the context of social innovations, where collaboration with local communities, non-governmental organisations, or public institutions is crucial [92], extraversion becomes a valuable tool. Micro-entrepreneurs with extraverted traits find it easier to establish collaborations, leading to better feedback collection, involving the local community in the innovation creation process, and gaining support for socially innovative projects.

Extraverts typically demonstrate cognitive flexibility and a willingness to take risks [56]. In the context of social innovations, which often require experimentation, bold steps, and an "outside the box" approach [120], these personality traits are extremely valuable. Micro-entrepreneurs with higher levels of extraversion are more inclined to take risks, translating into a greater readiness to experiment, implement unconventional solutions, and engage in innovative social actions.

Extraverts, by nature, are also active in seeking new ideas and stimuli from the environment [57]. This trait fosters an active search for inspiration, which is crucial in the innovation process [121]. Social innovations often stem from the ability to combine different concepts or identify innovative solutions. Micro-entrepreneurs with extraverted traits are more open to diverse stimuli, contributing to the creation of more creative and unique social innovations.

Implementing social innovations requires not only generating ideas but also effectively communicating their social value. Extraverts, thanks to their ability to communicate effectively and build enthusiasm [20], are more efficient in conveying socially innovative ideas. They can inspire others and garner community support, which is crucial for the success of such initiatives.

## 6. Conclusion

To sum up, it is worth returning to the research question: *Can the social innovations introduced by micro-entrepreneurs be influenced by their personality traits*?

Drawing from the Big Five model [31, 56] and the conceptualisation of innovation outlined in the Oslo Manual [4], the key finding suggests that for micro-entrepreneurs, three traits positively influence their social innovations. These attributes encompass openness to experience, conscientiousness, and extraversion.

Given the relatively enduring nature of personality traits [122], it is worth considering how to practically support and develop the personality traits of micro-entrepreneurs, such as openness to experience, conscientiousness, and extraversion, in the context of introducing social innovations. Firstly, suggesting the organisation of workshops and training sessions focused on developing skills related to openness to experience, conscientiousness, and extraversion. For example, sessions on creating innovative questions, enhancing observation skills, and building networks. Secondly, it is valuable to develop the practice of mentoring in micro-enterprises. Proposing mentors who possess strong personality traits associated with social innovation. Such mentors can share their experiences and provide practical advice on effectively utilising these traits in business. Thirdly, various networking events can be indicated–such as industry meetings or conferences–that enable micro-entrepreneurs to establish new connections. Active participation in such events develops extraverted skills and expands the circle of potential business partners. Finally, curiosity among micro-entrepreneurs can also be stimulated by encouraging them to explore new areas, technologies, and social trends.

In conclusion, it is also important to highlight the research limitations and potential future research directions. First, the results of the presented research focused on Polish micro-entrepreneurs. Therefore, it is worth considering whether the observed trends also apply to micro-entrepreneurs in other countries with different cultural and economic conditions.

Second, the personality traits were examined based on the Big Five model. However, it should be noted that, despite its widespread acceptance, there are other methods for assessing human personality. Hence, future research could explore alternative methods, such as the Myers-Briggs Type Indicator [123] or the HEXACO model [124].

Third, observing the rapid development of artificial intelligence (AI) based on machine learning (ML) [125, 126] and its impact on various spheres of human life (both professional and private), it is worth pointing out the need to expand research on its effects on micro-entrepreneurs in the process of implementing social innovations, e.g. in the area of ​​strengthening the influence of their personality traits on the success of these initiatives.

## Supporting information

S1 Checklist (DOCX)
